# A New Method for Nonlocal Means Image Denoising Using Multiple Images

**DOI:** 10.1371/journal.pone.0158664

**Published:** 2016-07-26

**Authors:** Xingzheng Wang, Haoqian Wang, Jiangfeng Yang, Yongbing Zhang

**Affiliations:** 1 Shenzhen Key Laboratory of Broadband Network & Multimedia, Graduate School at Shenzhen, Tsinghua University, Shenzhen, China; 2 Shenzhen Institute of Future Media Technology, Shenzhen, China; Wayne State University, UNITED STATES

## Abstract

The basic principle of nonlocal means is to denoise a pixel using the weighted average of the neighbourhood pixels, while the weight is decided by the similarity of these pixels. The key issue of the nonlocal means method is how to select similar patches and design the weight of them. There are two main contributions of this paper: The first contribution is that we use two images to denoise the pixel. These two noised images are with the same noise deviation. Instead of using only one image, we calculate the weight from two noised images. After the first denoising process, we get a pre-denoised image and a residual image. The second contribution is combining the nonlocal property between residual image and pre-denoised image. The improved nonlocal means method pays more attention on the similarity than the original one, which turns out to be very effective in eliminating gaussian noise. Experimental results with simulated data are provided.

## Introduction

Image denoising [1–10] is a low-level image processing tool, but it’s an important preprocessing tool for high-level vision tasks such as object recognition [11,12], image segmentation and remote sensing imaging. In the practical imaging system, there exists different kinds of noise. This paper only focus on the zero mean additive gaussian noise, which can be formulated as:

$$y_{i}=x_{i}+\eta_{i}$$ (1.1)

*y*_*i*_ is the *i*-th patch of the observed image *y*, *x*_*i*_ is the original patch with the *i*-th pixel at it’s center, and *η*_*i*_ is the independent additive gaussian white noise. The gaussian term can be used to simulate the noise generated by the intrinsic thermal or electronic fluctuations of the acquisition devices [13].

In image denoising, an image is often divided into many small patches which are repeatedly appear. We can remove the noise by taking advantages of the redundant information of patches while preserving the slight structure of images at the same time. By utilizing the redundant patches, the nonlocal means (NLM) image denoising method [14] could achieve impressive performance which be regarded as the most popular denoising method. The basic principle of nonlocal means is to denoise a pixel by averaging its local neighborhood pixels with the clues of similarities of the redundant patches. It has shown to be an effective image denoising technique. But the definition of similarities between the patch of the noisy pixel and its spatially local neighborhood patches in NLM is not strict, it’s just calculated by a block matching process.

Many researches devote to reduce the computational complexity [15,16] of NLM method. To achieve this, one important issue is to define and select similar patches, and many researchers have been focused on modifying the block matching process hence to find the true similar patches. In [17] and [18], a moment-based and rotation invariant feature representations have been applied for robust block matching by Ji et al and Zimmer et al respectively. Both of Kervrann et al [19] and Thaipanich et al [20] utilized the width of local window to represent different kinds of texture patterns, such as smooth and edge regions. The work in [21] analyze a rotationally invariant similarity for nonlocal image denoising. Among these researches, it is easy to see that finding ‘true similar’ patches are the critical part in patch-based denoising algorithms. But what is the ‘true’ similarity is not easy to define.

Since the accuracy of similarity computation is clearly affected by how rich the priori information of noised images is, we hereby apply multiple noised images hence to improve the performance of NLM method. First, we propose to use two noisy images to denoise pixels. The input to our system is two different noisy images but with the same noise deviation. Instead of only one image, we calculate the weight from two noisy images. Another important issue is how to design the weight of the similar patches. In the original nonlocal methods, every block within the scope of the search will be assigned a weight. Although the weight is very small, large numbers will reduce the denoising performance. So we redesign the similarity weight which rules out blocks with large difference. Third, the residual image implies a lot of edge information. We combine the nonlocal information of residual image and pre-denoised image to improve the denoising performance.

## Nonlocal Means Denoising

The basic principle of the nonlocal means denoising is to replace the noisy gray-value *I*(*i*) of pixel *i* with a weighted average of the gray-values of all the pixels on the image. Because it needs too much computation, it is more practical to average the pixels in a smaller scope. We denote the pixel to be denoised by *i* and the pixels in the neighborhood of *i* by *j* and use them to denoise *i*. The estimated value $\hat{I}(i)$ for a pixel *i* is computed based on the weighted average of the neighborhood pixels *j* around pixel *i*:

$$I(i)=\sum_{j\in N_{i}^{s}}w_{ij}I(j)$$ (1.2)

Where $N_{i}^{s}$ is the search window of size (2*n*+1)*(2*n*+1) centered at *i* and *w*_*ij*_ is the weight of two pixels *i* and *j* which is calculated depending on the similarity of their patches and is defined as:

$$w_{ij}=\frac{1}{Z_{i}}exp^{-\frac{|N^{d}(i)-N^{d}(j){|}_{2}^{2}}{h^{2}}}$$ (1.3)

where *Z*_*i*_ is a normalizing term, $Z_{i}=\sum_{j}w_{ij}$, and *h* acts as a filtering parameter. It controls the decay of the weights as a function of the Euclidean distances. This parameter is typically adjusted manually in the algorithm.

**Fig 1:**
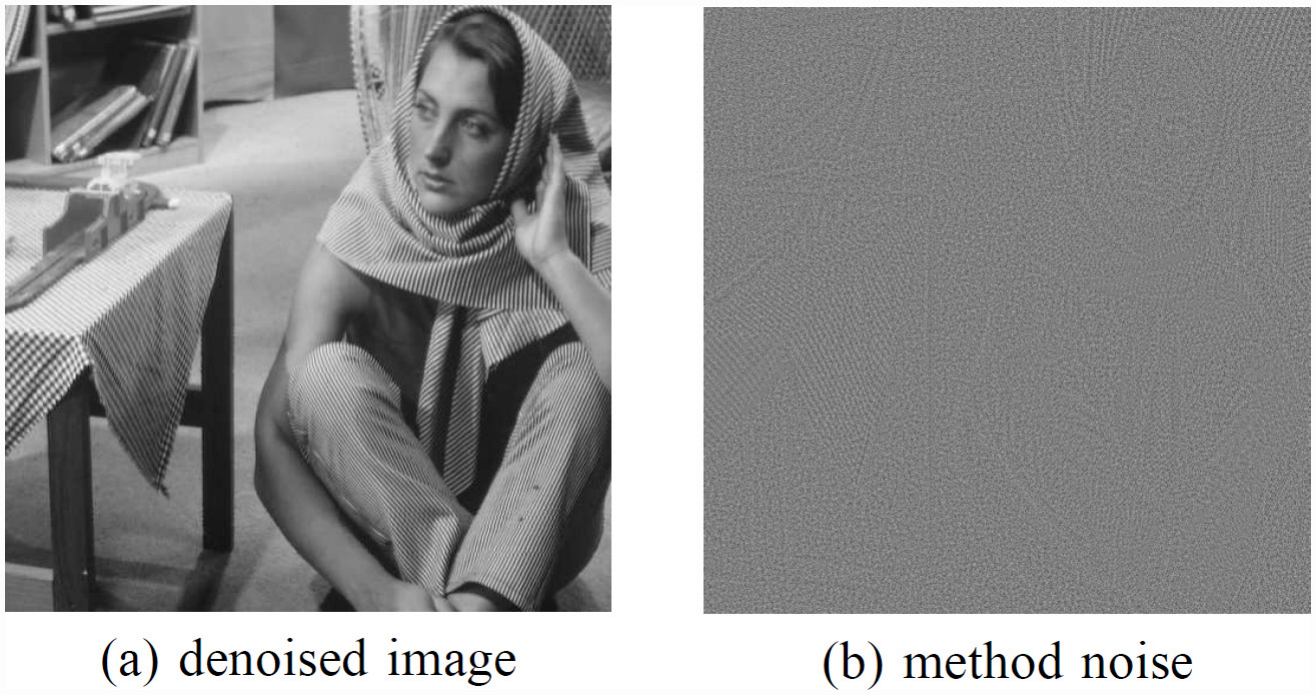
NLM denoising result under *δ* = 20. A denoised image. B residual image.

Fig 1A shows the original nonlocal means denoising result using the *w*_*ij*_ under the noise standard deviation *δ* = 20, and Fig 1B is the corresponding method noise, which appears obvious image structures that are similar with those in Fig 1A. The method noise here is defined as

$${\hat{n}}_{i}=y_{i}-{\hat{x}}_{i}$$ (1.4)

there exists some edge information in the residual image.

## Improved Nonlocal Means

**Fig 2:**
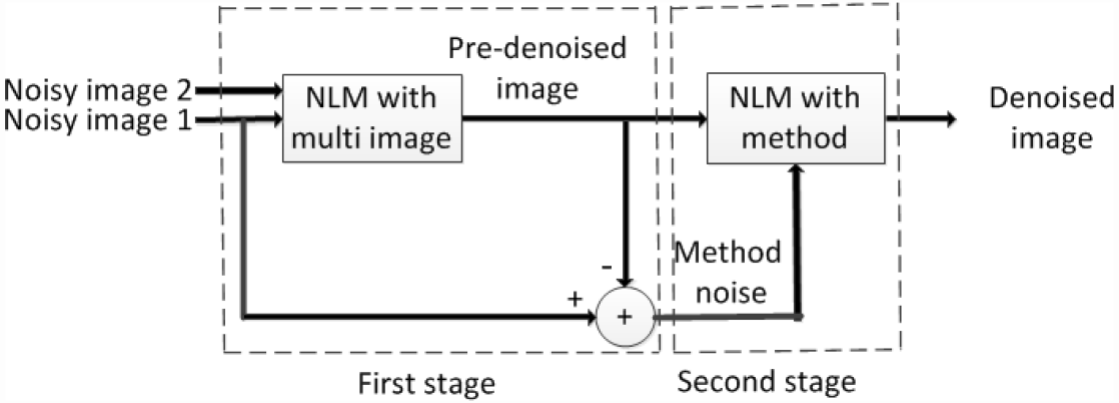
The flow chart of the improved NLM methods.

In this section, we will introduce the improved methods. In the first step we utilize the nonlocal information in two noisy images to improve the accuracy of similarity calculation. In the way, we can acquire the smoothed pre-denoised image and the residual image. In the second denoising step, we get the final denoised image by combining the nonlocal information in pre-denoised image and residual image. The flow chart is showed in Fig 2.

### Multi-Image

**Fig 3:**
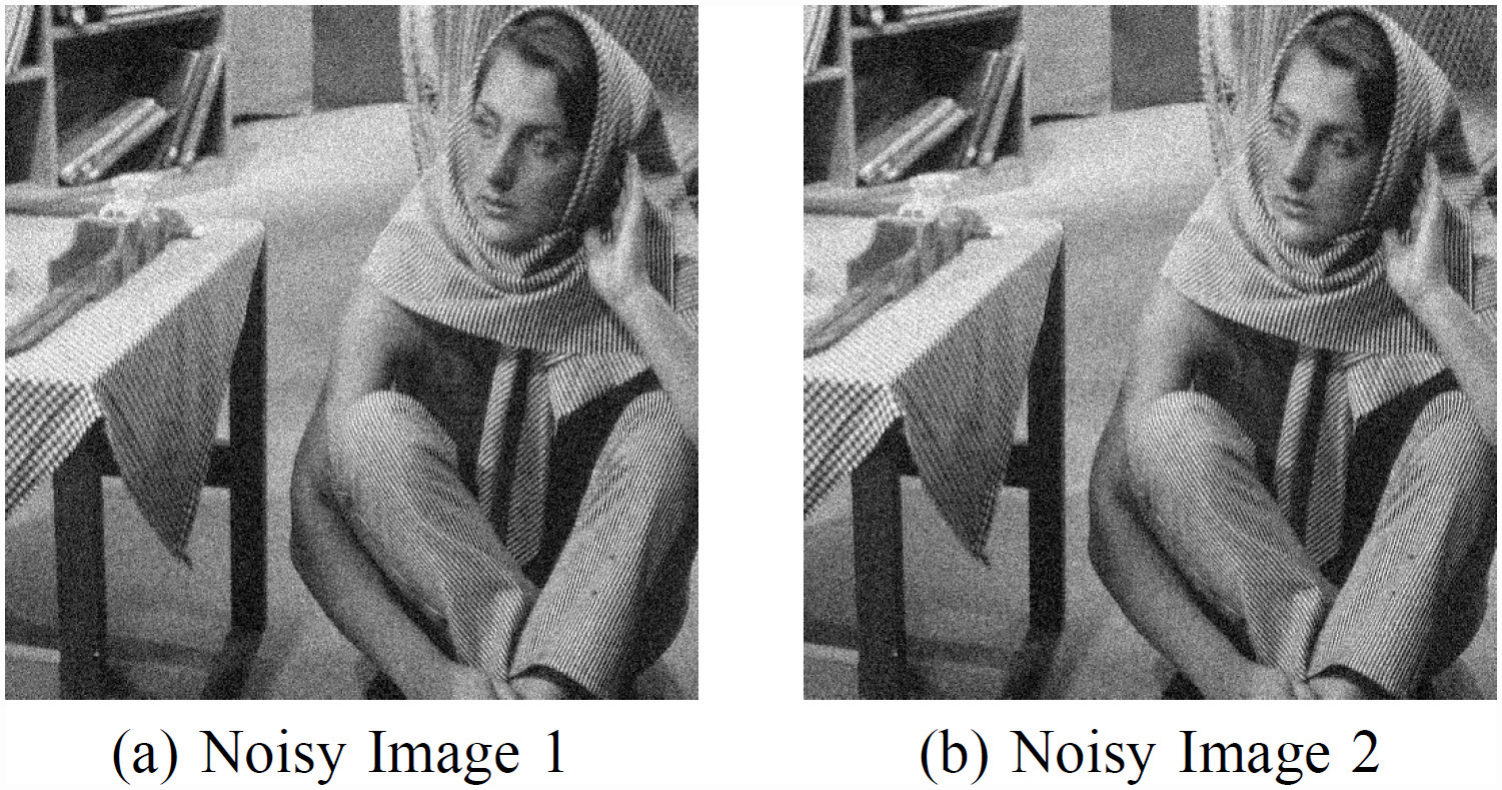
Barbara and two noisy images with noise δ = 20. A Noisy Image 1. B Noisy Image 2.

The nonlocal means algorithm utilizes similar blocks to denoise the point. In the original algorithm, polluted pixels are recovered from weighted average of the similar pixels. Many researches devoted to find the ‘true’ similar pixels. In this work, we try to solve this problem in another way, we calculate the weight from two noisy images but with same noise deviation. We add noise to I, the original clean image, and get Fig 3A and 3B, two different noisy images with same noise deviation, which is denoted by *I*_*1*_ and *I*_*2*_. In the real world, we could get such two images using the very fast camera with the continuous capture function.

We denote the pixel to be denoised in image *I*_1_ by *i*_1_ and the neighborhood pixels of *i*_1_ by *j*_1_. And we denote by *i*_2_ the pixel with the same position as *i*_1_ in *I*_2_ and the neighborhood pixels of *i*_2_ by *j*_2_. So the new weight between *i*_1_ and *j*_1_ is calculated by

$$w_{i_{1}j_{1}}=\frac{1}{Z_{i_{1}}}exp^{-\frac{|N^{d}(i_{1})-N^{d}(j_{1}){|}_{2}^{2}}{h_{1}^{2}}-\frac{|N^{d}(i_{2})-N^{d}(j_{2}){|}_{2}^{2}}{h_{2}^{2}}}$$ (1.5)

where $Z_{i_{1}}$ is a normalizing term, $Z_{i_{1}}=\sum_{j_{1}}w_{i_{1}j_{1}}$, *h*_1_ and *h*_2_ act as filtering parameters.

### Redesigning the weight

In the original nonlocal methods, every block within the search scope would be assigned a weight. Although the weights of the unsimilar blocks are very small, the large number of them will reduce the denoising performance. We redesign the similarity weight which rules out blocks with large difference in the same time. We denote the low limit by *ϵ*_1_ and the up limit by *ϵ*_2_. If $w_{i_{1}j_{1}}$ is less than *ϵ*_1_, the similarity of two blocks is small, so we set the weight $w_{i_{1}j_{1}}$ as 0 to rule out bad pixels. The redesigned weight is as follow:

$${\omega^{\prime }}_{i_{1}j_{1}}=\{\begin{array}{c} 0,\;\omega_{i_{1}j_{1}}<\varepsilon_{1}\\ \omega_{i_{1}j_{1}},\;{\frac{1}{Z^{\prime }}}_{i_{1}}\omega_{i_{1}j_{1}}<\varepsilon_{1} \end{array}$$ (1.6)

where ${Z^{\prime }}_{i_{1}}$ is the normalizing term, ${Z^{\prime }}_{i_{1}}=\sum {}_{j_{1}}{\omega^{\prime }}_{i_{1}j_{1}}$, With the new weight, we can calculate the estimated value of pixel *i*_1_ by

$${\hat{I}}_{1}=\sum_{j_{1}\in N_{i_{1}}^{s}}{\omega^{\prime }}_{i_{1}j_{1}}I_{1}(j_{1})$$ (1.7)

### Method noise

**Fig 4:**
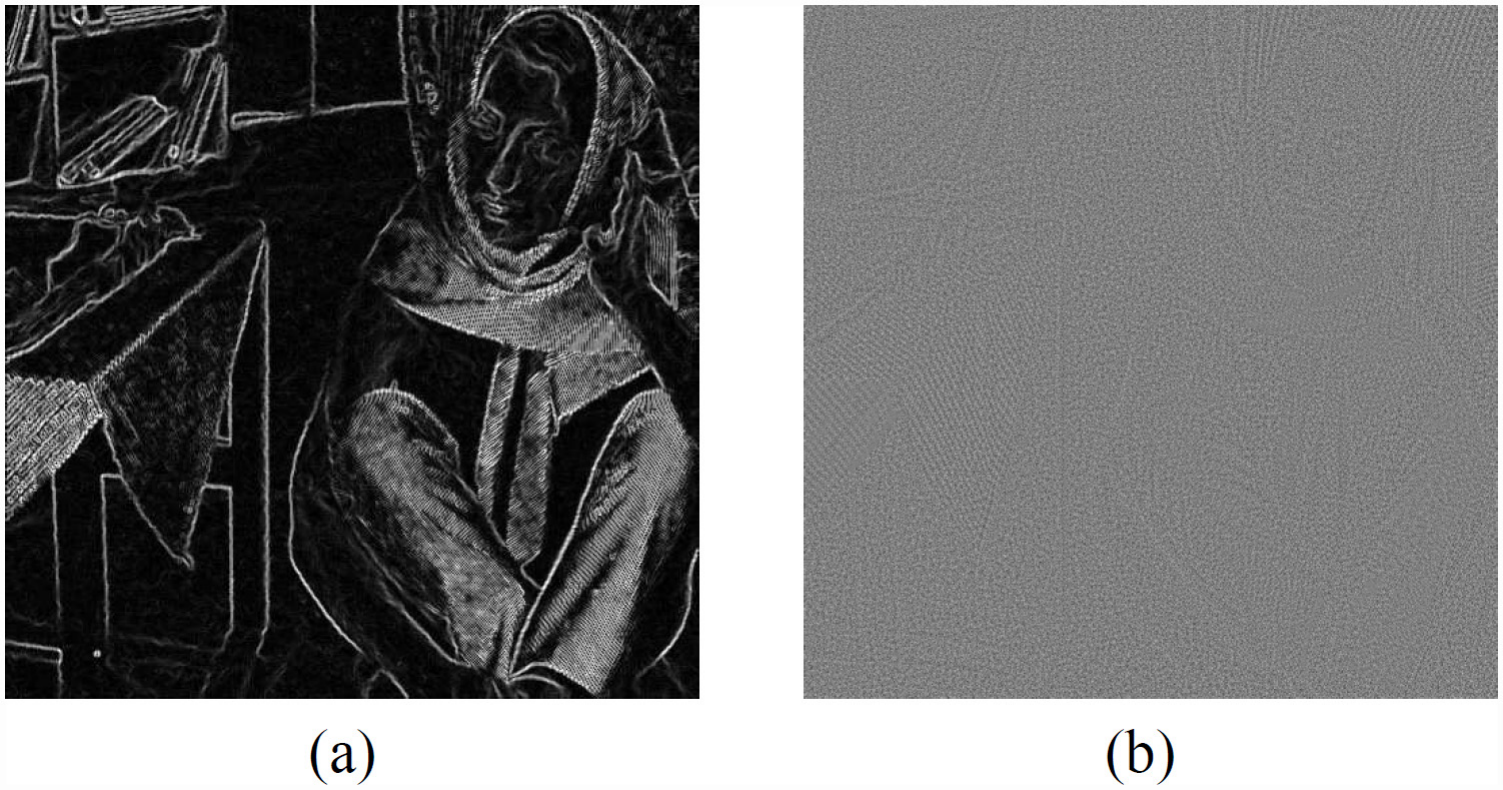
A is grad image and B is residual image.

In this subsection, we will show how to approximately estimate the similarities between different patches using residual image and pre-denoised image, and thus obtain the new weight. In the first denoising process, we got one residual image, shown in Fig 4B. In order to improve the accuracy of similarity calculation, we combine the similarity of the pre-denoised image and the residual image. According to the observation, we find that the residual image only provides abundant information in edge area. However, the flat area is largely covered by noise, without any useful information. So we should calculate the gradient image Fig 4A to determine the usage of residual nonlocal properties.

We denote the pixel in the pre-denoised image by *i*_*p*_, and denote the neighborhood pixels of *i*_*p*_ by *j*_*p*_. We then denote the pixel with same position as *i*_*p*_ in the residual image by *i*_*m*_, and denote the neighborhood pixels of *i*_2_ by *j*_*m*_. So the new weight between pixel *i*_1_ and *j*_1_ is calculated by

$$w_{ij}=\frac{1}{Z_{i}}exp^{-\frac{|N^{d}(i_{p})-N^{d}(j_{p}){|}_{2}^{2}}{h_{p}^{2}}-\frac{|N^{d}(i_{p})-N^{d}(j_{p}){|}_{2}^{2}}{h_{p}^{2}}}$$ (1.8)

where *Z*_*i*_ is a normalizing term,$Z_{i}=\sum_{j}w_{ij}$, *h*_*p*_ is used to control the attenuation of weight of pre-denoising image, and *h*_*m*_ is used to control the attenuation of weight of residual image. *h*_*p*_ and *h*_*m*_ will be adjusted in the experiment.

## Experiments

To validate the effectiveness of our method, we have used standard images such as Barbara, Boat, Fingerprint, House, Lena, Baboon with an additive white Gaussian noise (*δ*_*n*_ = 20 and *δ*_*n*_ = 10). We compare our approach to the well-known filtering techniques such as the bilateral filter [22], the traditional nonlocal means [14], the total variation [23] and the anisotropic diffusion [24]. The parameters of the methods should be tuned to get a good balance between texture preservation and noise suppression, measured by the peak signal noise ratio(PSNR). The parameters in our method for all experiments are as follows: *δ* = *δ*_*n*_ and a window size of 5*5.

In image denoising, the peak signal to noise ratio is often considered as the objective criteria. Given a noise-free m×n monochrome image I and its noisy approximation K, MSE and PSNR is defined as:

$$MSE=\frac{1}{mn}\sum_{i=0}^{m-1}\sum_{j=0}^{n-1}{\left[I(i,j)-K(i,j)\right]}^{2}$$ (1.9)

$$PSNR=10log_{10}\left(\frac{MAX_{I}^{2}}{MSE}\right)$$ (1.10)

Another image quality assessment index is the structural similarity (SSIM) index, which is based on the computation of three terms, namely the luminance term, the contrast term and the structural term, the overall index is a multiplicative combination of the three terms.

$$SSIM(i,j)={\left[I(i,j)\right]}^{\alpha }\cdot {\left[c(i,j)\right]}^{\beta }\cdot {\left[s(i,j)\right]}^{\gamma }$$ (1.11)

Where $I(i,j)=\frac{2\mu_{x}\mu_{y}+C_{1}}{\mu_{x}^{2}+\mu_{y}^{2}+C_{1}}$, $c(i,j)=\frac{2\sigma_{x}\sigma_{y}+C_{2}}{\sigma_{x}^{2}+\sigma_{y}^{2}+C_{2}}$, $s(i,j)=\frac{\sigma_{x}{}_{y}+C_{3}}{\sigma_{x}\sigma_{y}+C_{3}}$, and *μ*_*x*_, *μ*_*y*_, *σ*_*x*_, *σ*_*y*_ and *σ*_*xy*_ are the local means, standard deviations, and cross-covariance for images.

We show the PSNR and SSIM by different methods in Table 1, and it confirms that our method has better performances than the other methods. We can find that the proposed algorithm can improve 0.8dB than the original nonlocal means.

**Table 1:** Comparison of PSNR and SSIM.

	Barbara		Boat		Fingerprint		House		Lena		Baboon	
delta	20	10	20	10	20	10	20	10	20	10	20	10
TV	26.18	29.6	27.72	32.17	26.08	30.65	28.43	33.86	28.45	33.83	25.18)	-
	(0.84)	(0.88)	(0.85)	(0.92)	(0.84)	(0.89)	(0.87)	(0.93)	(0.87)	(0.94)	(0.83	
AD	26.45	30.85	28.06	31.92	24.81	29.02	29.41	33.72	29.27	33.36	23.68	-
	(0.84)	(0.89)	(0.86)	(0.91)	(0.82)	(0.88)	(0.88)	(0.92)	(0.88)	(0.94)	(0.82)	
Bilateral	26.75	31.05	27.82	31.52	24.12	28.81	29.18	33.4	29.28	33.01	24.95	29.31)
	(0.85)	(0.89)	(0.85)	(0.91)	(0.83)	(0.88)	(0.88)	(0.92)	(0.88)	(0.93)	(0.83)	(0.88
NLmean	28.78	32.96	28.92	32.49	26.45	30.6	30.86	34.66	31.13	34.65	25.18	29.54
	(0.87)	(0.92)	(0.88)	(0.92)	(0.84)	(0.90)	(0.90)	(0.94)	(0.92)	(0.94)	(0.84)	(0.88)
Multi	29.31	33.56	29.79	33.1	27.58	31.89	31.53	35.5	31.45	35.3	25.97	31.42
	(0.88)	(0.94)	(0.88)	(0.95)	(0.86)	(0.92)	(0.93)	(0.96)	(0.92)	(0.96)	(0.85)	(0.93)

**Fig 5:**
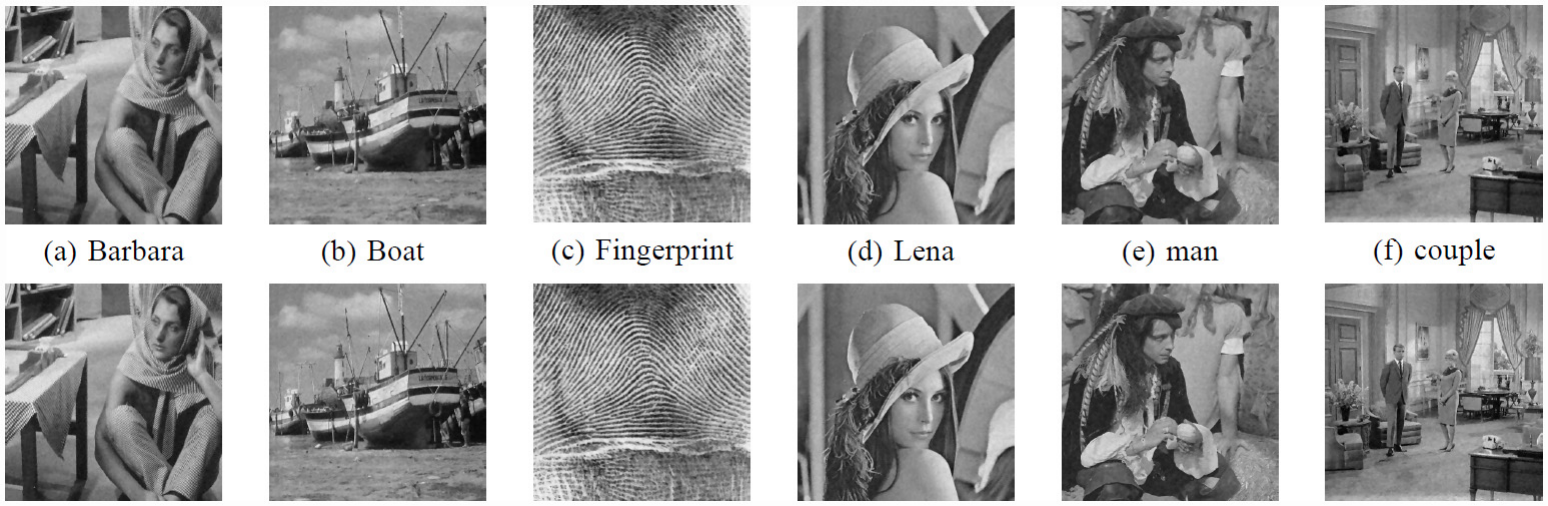
Comparison of different images with noise δ = 20. (a) Barbara. (b)Boat. (c) Fingerprint. (d) Lena. (e) man.

In Fig 5, we compared the denoising performance of 6 images, the above row is the result using the origin nonlocal means, the following row is result using the improved method.

**Fig 6:**
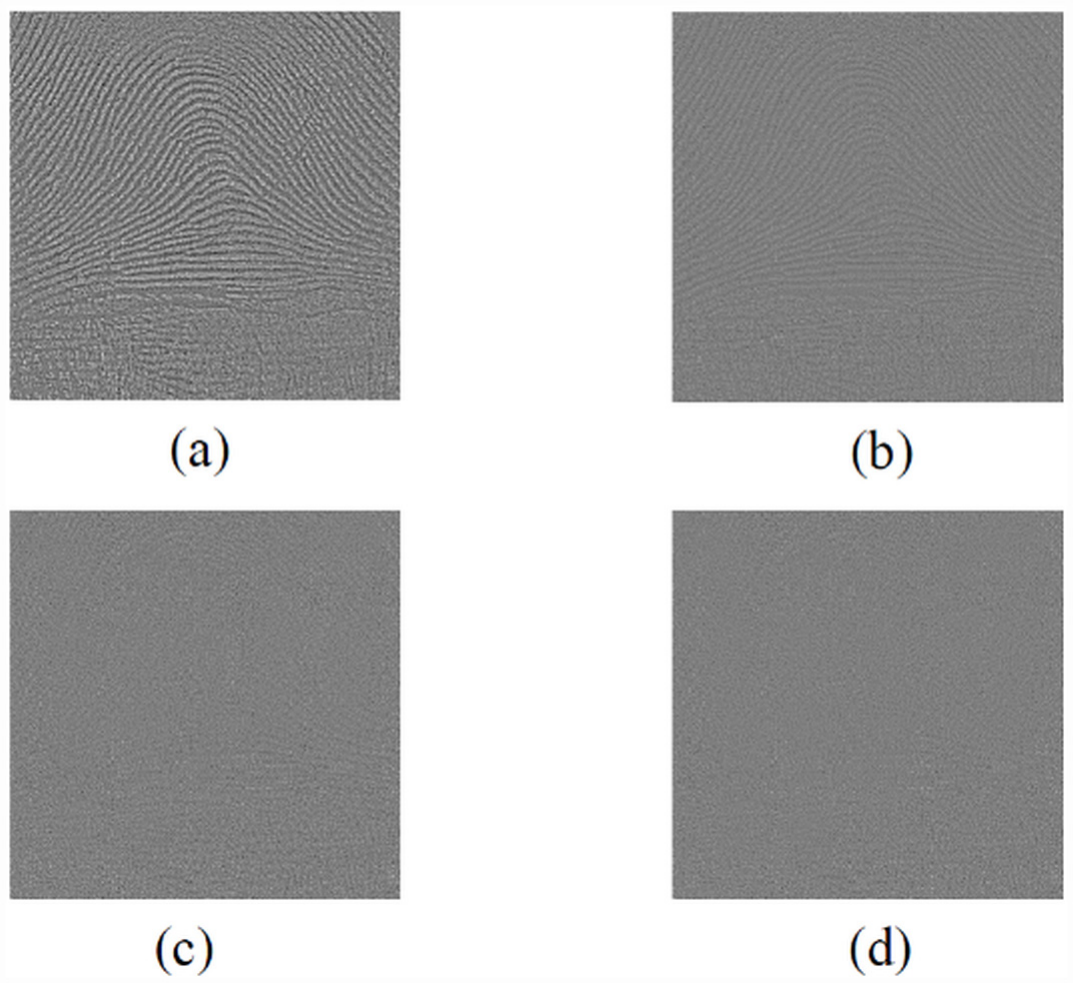
Comparison of residual image by different methods. (a) gaussian. (b) bilateral. (c) original NLM. (d) improved NLM.

In Fig 6, we show the residual of four methods, gaussian filter(a), bilateral filter(b), the origin nonlocal(c) and the improved nonlocal means(d). Although some image structure is still visible in the residual image of our method, the amount of remaining structure is much less than those using gaussian, bilateral and origin NLM.

## Conclusion

In this paper we redesign the weight in nonlocal image denoising. There are two main contributions of this paper. In the first denoising process, we add a noisy image of which the noise deviation is equal to that of the original noisy image. It can improve the accuracy of finding similar blocks by using the nonlocal property of two images. At the same time, we rule out the smaller weight blocks, thus reduce the interference of unsimilar blocks. In the second denoising step, it can further improve the accuracy by using the nonlocal similarity of the residual image. The experimental results show that the proposed algorithm can contribute an improvement of about 0.8 dB than the result of the original nonlocal means. As the speed of camera becoming faster, we will consider using multiple images in the camera denoising technology in the later study. It is also a good research direction to find how to better utilize the edge information in the residual image. However, since our methods need multiple images with the same noise deviation, the implementation of the image acquisition and the processing speed are limited and need to be further improved.
